# Quantity, Quality, and Performance of Corporate Social Responsibility Information Disclosure by Listed Enterprises in China: A Regional Perspective

**DOI:** 10.3390/ijerph17072245

**Published:** 2020-03-27

**Authors:** Haifeng Zhang, Zhuo Zhang, Adrian Tan, Ekaterina Steklova

**Affiliations:** 1 College of Economics and Management, Nanjing University of Aeronautics and Astronautics, Nanjing 211106, China; zhanghaifeng027@126.com; 2 Management Science, University of Waterloo, Waterloo, ON N2L 3G1, Canada; 3 Supply Chain Management, Penn State New Kensington, 3550 Seventh Street Road, New Kensington, PA 15068-1765, USA; 4 Tengfay Consulting, 64 Meadowlane Dr, Kitchener, ON N2N1E9, Canada; steklovakate@gmail.com

**Keywords:** regional, corporate social responsibility, information disclosure, corporate performance

## Abstract

The purpose of this article is to promote an increase in the number of enterprises that will disclose corporate social responsibility (CSR) information, and to improve on their quality of CSR information disclosure. Using the theory of organizational ecology, we propose that the density of companies that disclose CSR information in a region has an impact on both the quality and the performance of CSR disclosures. The study results suggest that an increase in the density of CSR information disclosing enterprises in a region will increase the number of enterprises with disclosure intentions. A density increase has a nonlinear influence on the quality of CSR information disclosure and on corporate performance, where the influence of disclosing enterprises’ density on corporate performance is partly mediated by the quality of CSR information disclosure. Our research also shows that the impact of density change of disclosing enterprises on the quality of CSR information disclosure is mediated by corporate capital structure.

## 1. Introduction

Enterprises engaging in socially responsible behaviors toward employees, consumers, environments, societies, and other stakeholders can help decrease the social gap between the rich and the poor, reduce environmental pollution, and mitigate climate change [1,2]. Corporate disclosure of social responsibility information can help enterprises establish a good social image, and further promote sustainable development. For these reasons, the topic of corporate social responsibility (CSR) information disclosure has increasingly come under the scrutiny of both enterprises and government agencies in China. In 2006, the State Grid Corporation of China issued the first CSR information report in China. From 2009 to 2018, an average of 48 additional enterprises follow suit to disclose CSR information each year. In 2018 itself, a total of 851 A-share listed enterprises in China disclosed CSR information reports. This represents an annual increase of 1.29 times in the number of disclosing enterprises over ten years. Rankins CSR Ratings (RKS), a company which specializes in CSR reporting and consulting in China [3], reports that the average rating score of these enterprises increased from 29.5 in 2009 to 42.5 in 2018. This improvement indicates that the quantity and quality of CSR information disclosures of listed enterprises in China have developed and matured accordingly.

Other scholars have studied the motivations of CSR information disclosure in terms of organization legalization, economic purpose, and stakeholder theory [1,4,5]. Among these, both corporate managers and academic theorists pay particular attention to economic motivation. Many scholars believe that CSR information disclosure behavior can improve the financial or corporate performance of enterprises [6] and improve enterprise value [7,8]. However, other scholars believe that CSR information disclosure either has a negative impact on corporate performance [9,10], or has no correlation to the improvement of corporate performance [4]. Equally inconsistent conclusions have emerged from the study of listed enterprises in China. Our literature review of Chinese-listed enterprises research found that scholars generally make two assumptions when discussing the economic value of CSR information disclosure behavior. First, it is supposed that the behavior and quality of CSR information disclosure are consistent actions arising from corporate decision-making. This assumes that the decision to provide disclosure of CSR information is biased with an intent to support a focal enterprise’s economic purposes. Second, based on the above assumption, the focal enterprise’s decision makers only consider the factors of the enterprise itself, and do not consider the interactive impact of the disclosure quality or disclosure intention of other enterprises in the region.

We wish to determine whether an increase in the number of disclosing enterprises in a region will affect the quality of CSR information disclosure and corporate performance. Financial data statistics of listed enterprises in China from 2009 to 2017 show that the Tobin Q value and total asset growth rate of enterprises that disclosed CSR information were significantly lower than those that did not disclose, while the asset–liability ratio, which is the ratio of total liabilities to total assets of an enterprise, is significantly higher among disclosing compared with non-disclosing enterprises. Please see the descriptive statistical analysis for specific data. In our analysis, we calculated the mean value of the number of disclosing enterprises per region, and sorted the 31 provinces and autonomous regions of China by this mean value so that regions that score higher than the mean value are defined as the high-density area, while regions scoring lower are defined as the low-density area. We find that the return on assets, and the quality of CSR information disclosure of the sampled enterprises in high-density regions were both higher than those in low-density regions. Based on the above analysis, we conclude that the disclosure intention of enterprises, and the quality of CSR information disclosure should be treated separately. This is because the initial intention of the enterprise’s decision to disclose CSR information is not aimed at economic benefits, but is to improve capital structure. After enterprises decide to disclose CSR information, the quality of CSR information disclosure results from enterprises’ decisions based on their comprehensive consideration of internal and external factors, and on the number of disclosing enterprises in the region. Improving the quality of CSR information disclosure in turn can influence corporate performance.

It is necessary to take the research a step further to clarify the mechanism by which disclosure density impacts the quality of disclosure and corporate performance of disclosing enterprises, because knowing this will significantly improve our understanding of how to increase the number of disclosing enterprises, and improve the quality of CSR information disclosure. We adopt perspectives from the theory of organizational ecology to consider the relationships resulting from CSR information disclosure density in a region, including the effects of mutual influence concerning CSR information disclosures among enterprises in a region, and the effects on CSR information disclosure quality and the corporate performance of those enterprises. There are three research contributions in this paper. First, it extends theoretical research on CSR information disclosure from a regional perspective. It explains the relationship between the number of disclosing enterprises in a region, the quality of CSR information disclosures, and corporate performance. The results provide a decision basis for senior management to improve their disclosure system, for managers to improve their disclosure performance, and for government agencies to craft relevant policies. Second, we construct a theoretical framework that relates the density of CSR information disclosure in a region to the quality of CSR information disclosure, and propose a two-phases theory that explains the behavior and the quality of CSR information disclosure among enterprises. Lastly, we wish to highlight that the value-added effect of CSR information disclosure to enterprises is an improvement to their capital structure. This is a contribution that is in line with the economic value perspective of CSR information disclosure theory.

The rest of this paper is organized as follows. Section 2 provides a theoretical analysis and presents the research hypotheses. Section 3 introduces the data, variables, and models for the empirical analysis. Section 4 presents and analyzes the empirical results. Finally, Section 5 presents the concluding remarks and recommendations.

## 2. Theoretical Analysis and Research Hypotheses

### 2.1. The Behavior and the Density of CSR Information Disclosing Enterprises in a Region

Powell (1983) proposed a new institutional theory to explain the phenomenon of convergence among organizations [11]. When members of an organization adopt certain behaviors that are recognized as praise-worthy by the organization, these members will obtain a “legitimacy” granted by the organization. When such “legitimized” behaviors become popularized throughout the organization, the phenomenon of “isomorphism” will appear. Institutional theory treats enterprises as economic units that operate within the contexts formed by institutions that affect their behavior and impose certain expectations on them [12]. Therefore, it becomes possible that enterprises operating in countries with institutional similarities will adopt homogeneous behaviors [13]. From the perspective of institutional theory, enterprises in a region can obtain the recognition of their stakeholders by disclosing CSR information and thus improve the legitimacy of these enterprises. Given an increasing density of disclosing enterprises in the region, the occurrence of reciprocal diffusion will further promote the intention of enterprises to disclose CSR information.

Certain enterprises in China, notably listed enterprises, are required by national policy to disclose CSR information, but other enterprises can choose whether to disclose such information. These enterprises will consider their own needs and advantages for disclosure, but also consider the behavior of other organizations in their region. According to Powell (1983), enterprises that choose not to disclose information will face three types of institutional isomorphism pressure: mandatory isomorphism force, imitative isomorphism force, and normative isomorphism force from China’s current national CSR information disclosure policy [11]. When enterprises find that the imitation of other enterprises’ disclosure decisions can gain them more legitimacy, increased compensation, and can even conceal internal faults, then their motivation to disclose CSR information will be strengthened. As the number of disclosing enterprises increase in the region, this will generate ethical and moral pressure on non-disclosing enterprises in the region, as these enterprises will be perceived by the public as lacking in social responsibility. Most enterprises normally seek to claim social values that are in line with their regional environment and to adopt codes of conduct that are widely accepted by the public. Therefore, when the number of disclosing enterprises in a region increases, this will generate regional isomorphic pressure on non-disclosing enterprises. Therefore, we propose the following hypothesis:

**Hypothesis** **1.**  *When the density of disclosing enterprises in a region is high, the intention of other enterprises in this region to also disclose will increase.*

From the point of view of competitiveness, the lower the density of disclosing enterprises in the region, the less will be the direct competition between disclosing enterprises. Reduced competition means that a disclosing enterprise finds it easier to obtain a good image [14], to improve the frequency of its media exposure [15], and to obtain support and resources from regional institutions such as banks, chambers of commerce, or government agencies. Such enterprises have an incentive to improve the quality of their CSR information disclosure to compete for institutional resources from these stakeholders. This is because enterprises can use the quality of CSR information disclosure as a signal to stakeholders [16]. However, as improvement to the quality of disclosure increases, it also increases the burden on enterprises to fulfill their social responsibilities, and may also result in the leakage of internal information to competitors to put these enterprises at a disadvantage. Therefore, these enterprises eventually develop the motivation to reduce the details of information disclosure to minimize such risks [17]. Therefore, we propose the following hypothesis:

**Hypothesis** **2.**  *The density of CSR information disclosure in the region has an inverted U-shaped relationship to the quality of CSR information disclosure.*

### 2.2. The Density and Performance of CSR Information Disclosure in the Region

From the perspective of organizational ecology, as the number of enterprises in a region generally changes dynamically from few to many, this change in population density has different influences on corporate performance. At the time of the first appearance of new organizational forms in a region, due to the lack of prior public exposure, an increase in the number of enterprises adopting a new organizational form can produce reciprocal diffusion, improve the external environment for these enterprises, improve the legitimacy of these enterprises, and thus contribute a positive impact on these enterprises’ economic performance [18]. Kim (2014) views CSR information disclosure as a tool for corporate governance [19]. In this perspective, disclosure can improve corporate governance, and help enterprises obtain the key resources needed for development. This will in turn bring economic compensation to enterprises and help improve their economic performance. The given amount of resources in any region is limited. With an increase in the number of enterprises in a region, increased competition among enterprises will act to prevent a continuous increase in the density of enterprises. As the density of disclosing enterprises increases in a region, enterprises that wish to continue competing for resources and to maintain competitive advantages through disclosure will need to further improve their levels of social responsibility fulfillment, which will increase their work burdens and reduce their performance [20,21]. Hong (2017) study the influence of incubator density change on incubator performance in a province and find that there is an inverted U-shaped relationship between incubator density and corporate performance [18]. This indicates that high organizational density in a region will have a negative impact on corporate performance. Therefore, we propose the following hypothesis:

**Hypothesis** **3.**  *An increase in the density of disclosing enterprises in a region will initially improve the corporate performance of enterprises, but as density continues to increase, it will negatively affect their performance such that the density and corporate performance of disclosing enterprises in a region has an inverted U-shaped relationship.*

The above analysis posits that the density of disclosing enterprises in the region affects the behavior of enterprises and the quality of their CSR information disclosures. Specifically, as the density of disclosing enterprises in a region increase, it will have an impact on the competitive and cooperative relationships among enterprises. These relationships are mainly reflected in term of the convenience of obtaining resources from the external environment. When the density of disclosing enterprises is low, it is easy for these enterprises to improve the quality of CSR information disclosure in order to maintain competitive optimization. Conversely, when the density is high, it becomes more difficult for the enterprises to compete for external resources. As such, the quality of CSR information disclosures and enterprises’ access to resources needed for development has a strong correlation. In other words, the influences of competition and cooperation among enterprises as impacted by the change to the density of disclosing enterprises in a region on performance may be largely due to the impact on the quality of CSR information disclosure, which further affects performance. Therefore, we propose the following hypothesis:

**Hypothesis** **4.**  *The quality of CSR information disclosure has a mediating effect on the relationship between the density of disclosing enterprises and corporate performance.*

### 2.3. The Intermediary Effect of Capital Structure

As stated above, the asset–liability ratio of disclosing enterprises is higher than that of non- disclosing enterprises, which indicates that the disclosure of CSR information can affect the capital structure of enterprises. Derrien (2016) finds that CSR information disclosure can alleviate corporate financing constraints [22]. Christine (1997), Lambert (2007), and Dhaliwal, et al. (2010) believe that CSR information disclosure could reduce corporate financing costs [7,23,24]. Goss (2011) find that the CSR fulfillment will increase the debt ratio of an enterprise [25], while Almazan (2004) find that a disclosing enterprise devotes more attention to risk management, which in turn is conducive to reducing the level of the enterprise’s operating leverage [26]. Yang (2015) studies the relationship between capital structure and performance in disclosing enterprises [27], and finds that when the disclosure quality is high, the capital structure is negatively correlated with corporate performance, while when the disclosure quality is low, the capital structure is positively correlated with corporate performance.

Data obtained from the RKS show that both the number of disclosing enterprises, and the quality of disclosures in China has been increasing year by year. This indicates that an increase in the number of disclosing enterprises in the region can promote an improvement to the quality of CSR information disclosure. However, improving the quality of CSR information disclosure will affect the capital structure of enterprises, and thus the corporate performance of enterprises. Therefore, we propose the following hypotheses:

**Hypothesis** **5.**  *The density of disclosing enterprises, by influencing the capital structure of enterprises, influences the quality of CSR information disclosure in a region.*

In other words, capital structure has a mediating effect between the relationship of the density of disclosing enterprises and the quality of CSR information disclosure in a region.

**Hypothesis** **6.**  *The density of disclosing enterprises, by influencing the capital structure of enterprises, influences the performance of CSR information disclosure in the region.*

In other words, capital structure has a mediating effect between the relationship of the density of disclosing enterprises and the performance of CSR information disclosure in a region.

**Figure 1:**
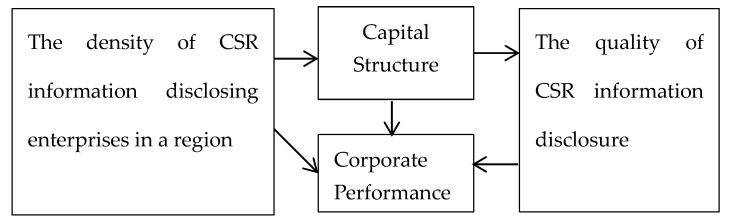
Theoretical model. CSR: corporate social responsibility.

Through the above analysis, the theoretical model of this paper is proposed as shown in Figure 1.

## 3. Data, Variables, and Models

### 3.1. Sample and Data

This study uses data from enterprises listed in China to research the relationship between the density, quality, and performance of CSR information disclosure in a region. Since 2008, the Shanghai stock exchange has required that enterprises with a Shanghai corporate governance board, foreign enterprises, and financial enterprises listed in the Shanghai exchange must disclose their CSR reports together with their annual reports. Other qualified listed enterprises are also encouraged to disclose their CSR reports. In 2009, RKS started to evaluate and grade enterprises in China that disclose CSR information. In our sample, we collect data from the years 2009 to 2017. In order to make the data more robust, we deleted the financial insurance industry because the industry follows different reporting requirements, ST-type enterprises, i.e., loss-making or otherwise atypical enterprises, because their financial data is recorded differently, and enterprises with incomplete data. According to the CSR information rating data of listed enterprises published by RKS, 835 enterprises disclosed CSR information reports from 2009 to 2017, though not all enterprises provide reports every year. We obtained 4,619 sample data observations of CSR information disclosure over these nine years. We also collected data from 19,538 Chinese listed enterprises, which comprise both disclosing as well as non-disclosing enterprises. The financial data of listed enterprises in this paper were obtained from the China Stock Market & Accounting Research database, and the quality of CSR information disclosure data were obtained from RKS. The main variables were winsorized tailed at 1% and 99%.

### 3.2. Variable and Model

Our dependent variables are corporate performance (TBQ) and whether a listed enterprise discloses a CSR information report. Our independent variables are the density of CSR information disclosing enterprises. Our mediating variables are the quality of CSR information disclosure, and corporate capital structure.

(1) Corporate performance (TBQ). Corporate performance generally reflects an enterprise’s ability to provide all corporate stakeholders, including shareholders, creditors, managerial staff, employees, and government agencies, satisfying returns under value-centered management in accordance to the rule of law. We follow Liu and Zhang to adopt the Tobin Q value of the enterprise as the proxy variable for corporate performance [2].

(2) Dummy variable to indicate if an enterprise discloses a CSR information report (sfpl). A disclosing enterprise is identified with the value of 1, and a non-disclosing enterprise is identified with the value of 0 based on the RKS data.

(3) The density of enterprises that disclose CSR information in a region (qymd). This paper adopts the measurement method of enterprises density used in the field of organizational ecology. Hannan and Freeman (1977) propose to use the count of units in the population as a measure of population density [28]. Greve (2002), and Hong (2017) all use the number of organizations in a region as the method to measure organizational density to study the impact of density change on organizational performance [21,29]. This paper adopts the view of these scholars and define the density of disclosing enterprises in a region as the number of A-Share listed enterprises in China that disclose CSR information reports.

(4) The quality of CSR information disclosure (shzrjx). The CSR information rating score issued by RSK is well recognized in academic circles in China [30]. We adopt it as the proxy variable of the quality of CSR information disclosure. This data consists of four parts, which respectively evaluate the integrity, content, technical, and industrial nature of the report that discloses CSR performance. This paper uses the total score after adding together these four scores.

(5) Corporate capital structure (zcfzl). The total asset–liability ratio, which is the ratio of total liabilities to total assets of an enterprise, in the financial reports of listed enterprises is used as the proxy variable of the corporate capital structure.

(6) Control variables. We control for the influence of the enterprise’s internal and external factors on the model by adopting enterprise size, number of employees, operating performance, operating leverage, enterprise growth, sustainable growth rate, property right attribute, marketization index, region, industry, and year as control variables [2,13]. The main variables used in this study are shown in Table 1.

We built Model 1, a logit regression model, to test the influence of the density of disclosing enterprises in a region on the intention of enterprises to disclose CSR information:

Model (1) $$Logit\;sfpl_{it}=\beta_{0}+\beta_{1}qymd_{it}+\beta_{2}con_{it}+\varepsilon_{it}$$

Referring to Baron (1986), the three-step method is adopted to verify the mediating effect of the quality of CSR information disclosure and the capital structure [32]. First, Model 2 is constructed to verify the impact of the density of disclosing enterprises in the region on performance; next, Model 3 is constructed to verify the influence of density on the quality of CSR information disclosure in the region; and Model 4 is constructed to test whether the quality of CSR information disclosure has an intermediary effect.

Model (2) $$shzrjx_{it}=\beta_{0}+\beta_{3}qymd_{it}+\beta_{4}qymd_{it}^{2}+\varepsilon_{it}$$

Model (3) $$TBQ_{it}=\beta_{0}+\beta_{5}qymd_{it}+\beta_{6}qymd_{it}^{2}+\varepsilon_{it}$$

Model (4) $$TBQ_{it}=\beta_{0}+\beta_{5}^{\prime }qymd_{it}+\beta_{6}^{\prime }qymd_{it}^{2}+\beta_{10}shzrjx_{it}+\beta_{11}shzrjx_{it}^{2}+\varepsilon_{it}$$

Model (5) $$zcfzl_{it}=\beta_{0}+\beta_{7}qymd_{it}+\beta_{8}qymd_{it}^{2}+\varepsilon_{it}$$

Model (6) $$shzrjx_{it}=\beta_{0}+\beta_{3}^{\prime }qymd_{it}+\beta_{4}^{\prime }qymd_{it}^{2}+\beta_{9}zcfzl_{it}+\varepsilon_{it}$$

Model (7) $$TBQ_{it}=\beta_{0}+\beta_{5}^{\prime \prime }qymd_{it}+\beta_{6}^{\prime }qymd_{it}^{2}+\beta_{9}^{\prime }zcfzl_{it}+\varepsilon_{it}$$

where $sfpl_{it}$ represents an enterprise in i province that discloses CSR information in t year; $qymd_{it}$ represents the number of listed disclosing enterprises in i province in t year; $TBQ_{it}$ is the corporate performance of enterprises in i province in t year; $shzrjx_{it}$ is the CSR information rating score of enterprises in i province published by RKS in t year; and ${zcfzl}_{it}$ is the corporate capital structure in i province in t year. Please refer to the Annex for a summation of the models.

With reference to Model 2 to Model 7, we found that the mediating effect of corporate capital structure between the density and the quality of CSR information disclosure in the region and the mediating effect of corporate capital structure between the density and performance of CSR information disclosure in the region were verified.

The correspondence between the econometric model, hypothesis, sample and variables is shown in Table A1.

## 4. Empirical Test

### 4.1. Descriptive Statistical Analysis

We use Stata14 as our statistical analysis software. First, the total sample of A-share listed enterprises in China from 2009 to 2017 is divided into a group of disclosing enterprises and a group of non-disclosing enterprises, and compared. The two groups were tested using the t-statistic and Wilicoxon rank-sum test, and the results are shown in Table 2. As can be seen from Table 2, disclosing enterprises in China only account for approximately 1/5 of the total sample, which indicates that disclosure intention among enterprises is not wide-spread in today’s environment. The TBQ value and the growth rate of total assets of the disclosing group are all lower than that of the non-disclosing group, but the asset–liability ratio of enterprises is far higher in the disclosing group. This suggests that the initial motivation of corporate decision-making to disclose CSR performance may not be to improve the value of the enterprise or obtain more growth opportunities, but rather to obtain more external financing.

We next divided our sample into two groups according to the density of disclosing enterprises in the region, and compared the two groups. The results as per Table 3 shows that the return on equity, the growth rate of total assets and the quality of CSR information disclosure of enterprises in the high-density environment group all measure higher than those in the low-density group. The enterprise value (TBQ) of enterprises in high-density areas measure higher than those in low-density areas, but the difference is not statistically significant. The above results indicate that the density of disclosing enterprises in a region affects the quality of CSR information disclosure, financial performance, and growth opportunities of enterprises.

Table 2 and Table 3 show that disclosure intention and the decision to set the level of quality of CSR information disclosure should not be regarded as uniformly consistent among enterprises. The initial motivation of corporate disclosure intention may not be to improve corporate performance, but to improve capital structure, or to obtain more external financing. When enterprises make decisions to disclose CSR information, the quality of their disclosures will be adjusted later according to the competitive state of their surrounding environment. At that time, improvements to their quality of CSR information disclosures will serve to improve the financial performances and the growth opportunities of the enterprise.

### 4.2. Correlation Analysis of Major Variables

Pearson correlation analysis of each variable is shown in Table 4. The correlation coefficients among all variables were less than 0.8. The basic assumption is that when the correlation coefficient between variables is greater than 0.8, it presupposed collinearity between the variables. We examined the inflation factor of each variable (VIF), and found that the average inflation factor score is 1.86, with highest score at 2.99. These results suggest that a regression analysis will not produce serious multicollinearity. We adopt the fixed effects model for all model regression analyses in this paper based on the results of the F test and Hausman test.

### 4.3. Regression Analysis

#### 4.3.1. The Behavior and the Density of Disclosing Enterprises in a Region

We first applied Model 1 using the total sample data with controls to examine the density of CSR information disclosing enterprises on enterprises’ disclosure intention. Considering that the density of disclosed enterprises in a region is determined by the number of disclosed enterprises, there may be an inverse causal relationship between the two. In this paper, the regression is carried out by using the lag period of the enterprise density of disclosure in the region and the enterprise disclosure intention. In the regression analysis, factors such as company size, number of employees, business performance, business leverage, enterprise growth, sustainable growth rate, property right attribute, marketization index, region, industry, and year were also controlled. Logit and probit analysis results were obtained as shown in Table 5. The coefficient of qymd is 0.5684 (p<0.01) in M1, which indicates that an increase in disclosed enterprises in a region will affect other enterprises’ disclosure intentions. In other words, the behavior of listed enterprises in disclosing CSR information will affect the decisions of other enterprises in the region on whether or not to disclose CSR information. This finding supports Hypothesis 1. In order to fully illustrate the density of CSR information disclosing enterprises’ impact on enterprises’ disclosure intention, we conducted regression analysis on whether enterprises made voluntary disclosure. The coefficient of qyqd is 0.1889 (p<0.05) in M2, which indicates that an increase of disclosed enterprises in a region will also increase their intention to voluntarily disclose. After replacing with the probit model, the qymd regression coefficient is 0.3335 (p<0.01) in M3. This finding again supports Hypothesis 1.

In Table 6, M4–M6 report the influence of the density of disclosing enterprises in a region on the quality of CSR information disclosure. M4 shows the influence of various control variables on the quality of CSR information disclosure. In M5, the qymd coefficient of the primary term is 0.0139, but it is not significant. In M6, the qymd coefficient of the primary term (−0.1015, p<0.01) and quadratic term (0.0201, p<0.01) are both significant at the level of 1%, indicating that the density of disclosing enterprises in a region has a U-shaped relationship with the quality of CSR information disclosure, which does not show support for Hypothesis 2.

Although Hypothesis 2 has not been shown to be supported, the empirical analysis does show that there is a non-linear relationship between the density of disclosing enterprises and the quality of CSR information disclosure in a region. At the time of writing, the density of CSR information disclosure in China is not high. The average density of disclosing enterprises in all provinces and cities is 41, with a median of 39, with the lowest value being 1, and with the highest value being 99. From the competitive perspective, when the density of disclosing enterprises is low, the competition among enterprises is not high. Therefore, an increase in the density of disclosing enterprises may not initially affect the disclosure behavior of other enterprises in the region, so that the qymd coefficient is not significant in M5. As the density continues to increase, the quadratic term of qymd coefficient becomes positive and significant as per M6. This indicates that the continued increase of regional density of disclosing enterprises has served to trigger competition among enterprises, and promote the improvement of CSR information disclosure quality. However, as stated earlier, at present, the density of disclosing enterprises in China has not reached such an inflection point, and competition among enterprises appears instead to inhibit the quality of CSR information disclosure.

#### 4.3.2. The Density and Performance of Disclosing Enterprises in a Region

In Table 6, M7-M9 report the quality of CSR information disclosure impact on the corporate performance of enterprises. It is observed that the coefficient of qymd in M8 is negative, but not significant. In M9, the coefficients of both the qymd primary (0.4068,p<0.05) and the quadratic terms (−0.073, p<0.01) are significant. To note, the significance of the quadratic term indicates that there is an inverted U-shaped relationship between the quality of CSR information disclosure and the corporate performance of enterprises. This provides support for Hypothesis 3.

Following Baron (1986), the three-step method is adopted to test the mediating effect of the quality of CSR information disclosure in the relationship between the density of disclosing enterprises and the corporate performance of enterprises in a region [32]. In Table 6, M8 and M9 indicate that the density of CSR information disclosure in a region has a significant effect on the quality and performance of disclosing enterprises. When shzrjx is put into M11 and M11, it is found that the coefficients of the qymd primary and quadratic terms are both significant at the 5% level. However, in M10 and M11, both the coefficients of the qymd primary and quadratic terms are less than the coefficient value in M9, indicating that the effect of the density of disclosing enterprises on the corporate performance of enterprises in a region is partially mediated by the quality of CSR information disclosure. This supports Hypothesis 4.

#### 4.3.3. The Intermediary Effect of Capital Structure

Table 7 reports the mediating effect of corporate capital structure in the relationship among the density, quality, and performance of disclosing enterprises in a region. M12 and M13 report the impact of the density of disclosing enterprises in a region on the capital structure of enterprises, and find that an increase in the density of disclosing enterprises in a region is conducive to improving the capital structure of enterprises. M14 is based on the sample and variables found in M6, with the addition of the intermediate variable capital structure. We find that both the coefficients of the qymd primary and quadratic terms are significant at the 1% level. However, the coefficients of the qymd primary and quadratic terms in M14 are less than the coefficient value in M6, indicating that the effect of disclosing enterprise density on the quality of CSR information disclosure in a region is partially mediated by the corporate capital structure. This supports Hypothesis 5. M15 is based on M9, with the addition of the intermediate variable capital structure. We find that the coefficient of the qymd primary term is significant at the 5% level, and that the quadratic term is significant at the 1% level. But the coefficients of the qymd primary and quadratic terms in M15 are less than the coefficient in M9, indicating that the effect of disclosing enterprise density on performance in the region is partially mediated by the corporate capital structure. This provides support for Hypothesis 6.

## 5. Conclusions

This paper studies the impact of CSR information disclosure on the corporate performance of enterprises from a regional perspective. We find that changes to the density of disclosing enterprises will affect the quality and performance of CSR information disclosure in a region, and that the quality of CSR information disclosure plays a mediating role in the influence of the density of disclosing enterprises in a region on the corporate performance of enterprises. The research shows that the density of disclosing enterprises in a region mainly affects the quality of CSR information disclosure and corporate performance by influencing corporate capital structure. The research conclusions of this paper are:

(1) When studying the motivation of enterprises to disclose CSR information, especially for economic purposes, the disclosure decision of enterprises should be treated separately from the quality of CSR information disclosure. According to our statistical findings, it can be seen that the corporate performance of disclosing enterprises is lower than that of the non-disclosing enterprises. However, when examining only disclosing enterprises, the financial performance and growth capacity of enterprises in regions with high disclosure density are significantly better than those in low disclosure density regions. This indicates that when enterprises first take the decision to disclose CSR information, it is not strictly for economic purposes. It is only after the decision is made that enterprises use CSR information disclosure as a form of signaling to stakeholders to maximize benefits and improve the capital structure of the enterprises [33].

(2) The density of disclosing enterprises in a region has an impact on the quality of CSR information disclosure, indicating that the disclosing enterprises in a region mutually influence one another. While an increase in the density of disclosing enterprises in a region promotes improvement to the quality of CSR information disclosure, the improvement of the quality of CSR information disclosure itself increases the burden on enterprises and inhibits corporate performance. The influence of density on corporate performance is partly mediated by the quality of CSR information disclosure.

(3) There is a positive correlation between the quality of CSR information disclosure and corporate capital structure. The influence of density on CSR information disclosure and corporate performance is mediated by corporate capital structure.

The practical significance of this paper is as follows: first, from the regional perspective, the number of enterprises disclosing CSR information in the provinces, cities and autonomous regions of China still only accounts for a small proportion of listed enterprises, and the negative impact of the density of disclosing enterprises on the quality of CSR information disclosure has not reached an inflection point. Government agencies should help encourage enterprises within their jurisdictions to disclose CSR information to create better sustainable environments, support more growth opportunities, and alleviate any financial constraints for enterprises with bank credit and other financing channels. Second, from the CSR information disclosure intention perspective, one of the main motives in disclosure is to improve the corporate capital structure. The incremental value-added effect of CSR information disclosure is the maximization of benefits by dynamically adjusting the quality of CSR information disclosure after an enterprise improves its capital structure. An added implication is the cost of CSR information disclosure or corporate capital structure improvement. Managers need to seek to reduce the financing cost of enterprises to reduce the cost of CSR fulfillment, and thereby to improve the quality of CSR fulfillment in enterprises. Corporate managers should note that although CSR information disclosure may not be undertaken in the first place with the purpose of pursuing an economic benefit, the positive effects of CSR information disclosure include optimizing the capital structure, and improving corporate governance and performance. Corporate managers need to give full play to these positive effects of CSR information disclosure by consciously linking them with one another while at the same time gradually standardizing and improving the quality of CSR performance through an optimization of their governance structure. This balance is needed to form a healthy and sustainable interaction mechanism to prevent excessive pursuit of disclosure quality that can negatively affect corporate performance. This study has limitations that needed to be addressed in future research. For instance, private enterprises and state-owned enterprises found in China’s unique economic system occupy different market positions. New and interesting findings may emerge if these private enterprises and state-owned enterprises are analyzed separately. We intend to perform future research on the relationship between the density, quality, and performance of disclosing enterprises in a region from the perspective of private enterprises.

## Figures and Tables

**Table 1:** Main variables.

Name of Variable	Calculation of Variable
Corporate performance (TBQ)	Total asset market value/Total asset replacement cost
Dummy variable to indicate if an enterprise discloses a CSR report (sfpl)	A disclosing enterprise is identified with the value of 1, and a non-disclosing enterprise is identified with the value of 0 If disclosure the value is 1, otherwise it is 0, by the based on the RKS data
The density of disclosing enterprises in the region (qymd)	Logarithm of the number of disclosing enterprises in the region
The quality of CSR information disclosure (shzrjx)	Logarithm of the CSR information rating score as issued by Rankins CSR Ratings
corporate capital structure(zcfzl)	Total liabilities/total assets of an enterprise
Dummy variable of Ownership (gqxz)	State-owned enterprises are set to 1, others are set to 0
Enterprise scale (zczj)	Logarithm of total assets of an enterprise
Number of employees (people)	Total number of employees of an enterprise
Growth rate of total assets (grow)	Growth measured as ratio of end-of-year assets against start-of-year assets
Return on equity (roe)	Net profit/Net assets of an enterprise
Marketability index (schzs)	The annual marketization total index of each Chinese province as compiled by X L. Wang, et al. (2018) [31].
Operating leverage(lever)	Change rate of ebitda/change rate of production and sales volume
Sustainable growth rate (sust)	Return on equity * retention rate/(1- roe x retention rate)
Dummy variable of Region (dq)	The eastern coastal provinces of China are set to 1, and other provinces are set to 0
Dummy variable of Year (year)	Year dummy variable
Dummy variable Industry (hydm)	Industry classification and code of national economy of China (GB/T 4754-2017)

**Table 2:** Comparison of non-disclosing and disclosing groups.

Total Sample								
Variable	Non-Disclosing Enterprises			Disclosing Enterprises			Comparison Test	
	N	Median1	Mean1	N	Median1	Mean1	Chi2	MeanDiff
TBQ	14919	1.765	2.189	4619	1.511	1.891	235.304 ***	0.298 ***
qymd	14919	37	39.28	4619	41	43.42	62.002 ***	−4.138 ***
grow	14919	0.138	0.305	4619	0.109	0.160	82.983 ***	0.145 ***
roe	14919	0.074	0.083	4619	0.086	0.095	79.342 ***	−0.012 ***
zczj	14919	21.62	21.73	4619	22.96	23.07	2214.91 ***	−1.338 ***
zcfzl	14919	0.382	0.397	4619	0.503	0.491	590.371 ***	−0.094 ***
lever	14919	1.324	1.559	4619	1.358	1.598	25.010 ***	−0.039 ***
people	14919	7.345	7.353	4619	8.346	8.438	1387.8 ***	−1.085 ***
sust	14919	0.054	0.078	4619	0.063	0.079	60.255 ***	−0.001

*** represents 1% significance level.

**Table 3:** Comparison between density groups among disclosing enterprises.

CSR Information Disclosing Enterprises								
Variable	Low Density			High Density			Comparison Test	
	N	Median1	Mean1	N	Median1	Mean1	Chi2	MeanDiff
TBQ	2050	1.508	1.884	2569	1.514	1.897	0.08	−0.013
qymd	2050	15	18.20	2569	61	63.55	2904.023 ***	−45.350 ***
grow	2050	0.100	0.148	2569	0.116	0.170	14.270 ***	−0.023 ***
roe	2050	0.076	0.089	2569	0.092	0.100	42.279 ***	−0.011 ***
zczj	2050	22.95	22.99	2569	22.97	23.13	0.212	−0.143 ***
zcfzl	2050	0.494	0.488	2569	0.509	0.493	3.111 *	−0.004
shzrjx	2050	34.72	36.58	2569	36.23	39.32	21.284 ***	−2.737 ***
lever	2050	1.419	1.678	2569	1.315	1.534	76.039 ***	0.144 ***
people	2050	8.389	8.473	2569	8.319	8.410	1.165	0.063
sust	2050	0.057	0.073	2569	0.069	0.084	28.910 ***	−0.011 ***

*** represents 1% significance level, * represents 10% significance level.

**Table 4:** Correlation analysis of major variables.

Variable	TBQ	shzrjx	qymd	grow	roe	zczj	zcfzl	lever	people	sust	schzs
TBQ	1										
shzrjx	–0.121 ***	1									
qymd	0.044 ***	0.237 ***	1								
grow	–0.052 ***	–0.040 ***	0.054 ***	1							
roe	0.148 ***	0.025 *	0.054 ***	0.090 ***	1						
zczj	–0.400 ***	0.447 ***	0.062 ***	–0.095 ***	0.114 ***	1					
zcfzl	–0.275 ***	0.101 ***	–0.079 ***	–0.162 ***	0.009	0.555 ***	1				
lever	–0.006	0.056 ***	–0.105 ***	–0.193 ***	−0.496 ***	0.009	0.071 ***	1			
people	–0.273 ***	0.421 ***	–0.007	–0.126 ***	0.116 ***	0.689 ***	0.346 ***	0.121 ***	1		
sust	0.056 ***	–0.013	–0.002	0.006	0.127 ***	−0.002	0.031 ***	−0.041 ***	−0.007	1	
schzs	0.002	0.210 ***	0.708 ***	–0.000	0.036 **	0.098 ***	−0.040 ***	−0.086 ***	−0.015	0.009	1
VIF	Mean1.86	1.39	2.06	1.05	2.12	2.99	1.55	1.47	2.24	1.63	2.06

*** represents 1% significance level, ** represents 5% significance level, * represents 10% significance level.

**Table 5:** Logit regression analysis.

Variable	sfpl		
	M1	M2	M3
L.qymd	0.5684 ***	0.1889 **	0.3335 ***
	(0.0408)	(0.0901)	(0.0231)
roe	5.6984 ***	−3.1683 **	3.0396 ***
	(0.7278)	(1.5522)	(0.3886)
lever	0.0605 *	−0.0815	0.0404 **
	(0.0364)	(0.0728)	(0.0205)
zczj	0.8599 ***	−0.6666 ***	0.4973 ***
	(0.0308)	(0.0678)	(0.0175)
people	0.0703 ***	−0.1745 ***	0.0437 ***
	(0.0271)	(0.0624)	(0.0157)
sust	−3.6429 ***	1.562	−1.7974 ***
	(0.6208)	(1.4898)	(0.3103)
gqxz	0.6469 ***	−1.0279 ***	0.3779 ***
	(0.0490)	(0.1016)	(0.0283)
grow	−0.8772 ***	0.9124 ***	−0.4922 ***
	(0.0950)	(0.2140)	(0.0507)
schzs	−0.1424 ***	−0.1848 ***	−0.0878 ***
	(0.0197)	(0.0422)	(0.0113)
dq	yes	yes	yes
year	yes	yes	yes
hydm	yes	yes	yes
_cons	−21.8619 ***	17.0280 ***	−12.6606 ***
	(0.6235)	(1.3501)	(0.3488)
N	14877	2949	14877
LR chi2	3975.19	930.3	3972.53
Pseudo R2	0.227	0.239	0.227

*** represents 1% significance level, ** represents 5% significance level, * represents 10% significance level.

**Table 6:** The relationship between the density, quality, and performance of disclosing enterprises in a region.

Variable	shzrjx				TBQ			
	M4	M5	M6	M7	M8	M9	M10	M11
qymd		0.0139	−0.1015 ***		−0.0125	0.4068 **	0.4066 **	0.4043 **
		(0.0175)	(0.0335)		(0.0833)	(0.1593)	(0.1595)	(0.1598)
qymd2			0.0201 ***			−0.0730 ***	−0.0730 ***	−0.0726 ***
			(0.0050)			(0.0237)	(0.0237)	(0.0238)
shzrjx							−0.002	0.245
							(0.0777)	(0.8583)
shzrjx2								−0.0349
								(0.1209)
roe	2.8454 ***	2.8458 ***	2.8602 ***	−0.0343	−0.0347	−0.0387	2.8602 ***	2.8607 ***
	(0.2924)	(0.2924)	(0.2921)	(0.0615)	(0.0615)	(0.0614)	(0.2922)	(0.2922)
lever	0.0490 **	0.0491 **	0.0498 **	0.00250	0.00240	0.00220	0.0498 **	0.0498 **
	(0.0199)	(0.0199)	(0.0199)	(0.0042)	(0.0042)	(0.0042)	(0.0199)	(0.0199)
zczj	−0.6790 ***	−0.6795 ***	−0.6787 ***	0.0539 ***	0.0544 ***	0.0542 ***	−0.6786 ***	−0.6786 ***
	(0.0439)	(0.0440)	(0.0440)	(0.0092)	(0.0093)	(0.0092)	(0.0442)	(0.0442)
people	0.0759 ***	0.0760 ***	0.0792 ***	−0.00540	−0.00550	−0.00640	0.0792 ***	0.0793 ***
	(0.0287)	(0.0288)	(0.0287)	(0.0060)	(0.0060)	(0.0060)	(0.0288)	(0.0288)
sust	0.8811 ***	0.8810 ***	0.8773 ***	−0.00560	−0.00550	−0.00450	0.8772 ***	0.8765 ***
	(0.1190)	(0.1190)	(0.1189)	(0.0250)	(0.0250)	(0.0250)	(0.1189)	(0.1189)
gqxz	−0.4289 ***	−0.4286 ***	−0.4354 ***	0.0150	0.0146	0.0165	−0.4354 ***	−0.4359 ***
	(0.1177)	(0.1177)	(0.1176)	(0.0248)	(0.0248)	(0.0247)	(0.1176)	(0.1177)
grow	0.1669 ***	0.1671 ***	0.1667 ***	0.00200	0.00180	0.00190	0.1667 ***	0.1662 ***
	(0.0493)	(0.0493)	(0.0492)	(0.0104)	(0.0104)	(0.0103)	(0.0492)	(0.0493)
schzs	0.0655 **	0.0655 **	0.0897 ***	−0.000800	−0.000900	−0.00750	0.0897 ***	0.0894 ***
	(0.0285)	(0.0285)	(0.0296)	(0.0060)	(0.0060)	(0.0062)	(0.0296)	(0.0296)
dq	yes	yes	yes	yes	yes	yes	yes	yes
year	yes	yes	yes	yes	yes	yes	yes	yes
hydm	yes	yes	yes	yes	yes	yes	yes	yes
_cons	1.8962 ***	1.8664 ***	2.0701 ***	16.2836 ***	16.3105 ***	15.5695 ***	15.5736 ***	15.1480 ***
	(0.2164)	(0.2197)	(0.2250)	(1.0293)	(1.0450)	(1.0710)	(1.0832)	(1.8284)
N	4619	4619	4619	4619	4619	4619	4619	4619
r2	0.852	0.852	0.852	0.767	0.767	0.768	0.768	0.768
r2_a	0.817	0.817	0.818	0.714	0.714	0.714	0.714	0.714
F	113.3	109.7	107.2	49.72	48.11	47.01	45.57	44.22

*** represents 1% significance level, ** represents 5% significance level.

**Table 7:** Analysis of intermediary effect of corporate capital structure.

Variable	zcfzl		shzrjx	TBQ
	M12	M13	M14	M15
qymd	0.0154 *	0.0200	−0.1001 ***	0.3992 **
	(0.0093)	(0.0178)	(0.0335)	(0.1592)
qymd2		−0.0008	0.0200 ***	−0.0727 ***
		(0.0026)	(0.0050)	(0.0236)
zcfzl			−0.0675 **	0.3825 ***
			(0.0307)	(0.1460)
roe	−0.0425	−0.0424	−0.0416	2.8764 ***
	(0.0326)	(0.0326)	(0.0614)	(0.2920)
sust	0.0796 ***	0.0796 ***	0.0009	0.8468 ***
	(0.0133)	(0.0133)	(0.0251)	(0.1193)
gqxz	0.0395 ***	0.0394 ***	0.0192	−0.4505 ***
	(0.0131)	(0.0131)	(0.0247)	(0.1177)
zczj	0.1026 ***	0.1026 ***	0.0611 ***	−0.7180 ***
	(0.0049)	(0.0049)	(0.0098)	(0.0464)
schzs	−0.0007	−0.0004	−0.0075	0.0899 ***
	(0.0032)	(0.0033)	(0.0062)	(0.0295)
lever	0.0104 ***	0.0104 ***	0.00290	0.0459 **
	(0.0022)	(0.0022)	(0.0042)	(0.0199)
people	0.0006	0.0006	−0.0064	0.0789 ***
	(0.0032)	(0.0032)	(0.0060)	(0.0287)
grow	−0.0208 ***	−0.0208 ***	0.0005	0.1747 ***
	(0.0055)	(0.0055)	(0.0104)	(0.0493)
dq	yes	yes	yes	yes
year	yes	yes	yes	yes
hydm	yes	yes	yes	yes
_cons	−1.8915 ***	−1.9004 ***	2.2043 ***	16.1122 ***
	(0.1108)	(0.1146)	(0.2233)	(1.0625)
N	4619	4619	4619	4619
r2	0.900	0.900	0.852	0.768
r2_a	0.877	0.877	0.818	0.715
F	20.55	19.90	104.2	45.86

*** represents 1% significance level, ** represents 5% significance level, * represents 10% significance level.

## Appendix A

**Table A1:** Regression model, sample, and variable correspondence table.

Sort	Model	Sample	Dependent Variables	Independent Variables
M1	Model1	Total sample	sfpl	qymd
M2	Model1	Total sample	sfpl	qymd
M3	Model1	Total sample	sfpl	qymd
M4	Model2	Disclosing sample	shzrjx	Control variables
M5	Model2	Disclosing sample	shzrjx	qymd
M6	Model2	Disclosing sample	shzrjx	qymd\qymd2
M7	Model3	Disclosing sample	TBQ	Control variables
M8	Model3	Disclosing sample	TBQ	qymd
M9	Model3	Disclosing sample	TBQ	qymd\qymd2
M10	Model4	Disclosing sample	TBQ	qymd\shzrjx
M11	Model4	Disclosing sample	TBQ	qymd\qymd2\ shzrjx\ shzrjx2
M12	Model5	Disclosing sample	zcfzl	qymd
M13	Model5	Disclosing sample	zcfzl	qymd\qymd2
M14	Model6	Disclosing sample	shzrjx	qymd\qymd2\zcfzl
M15	Model7	Disclosing sample	TBQ	qymd\qymd2\zcfzl
